# Priorities and Future Actions for an Effective Use of Phytotherapy in Livestock—Outputs from an Expert Workshop

**DOI:** 10.3389/fvets.2017.00248

**Published:** 2018-01-22

**Authors:** Isabel Blanco-Penedo, César Fernández González, Lena-Mari Tamminen, Albert Sundrum, Ulf Emanuelson

**Affiliations:** ^1^Division of Ruminant Medicine and Veterinary Epidemiology, Department of Clinical Sciences, Swedish University of Agricultural Sciences, Uppsala, Sweden; ^2^Animal Welfare Subprogram, Institute of Agrifood Research and Technology, Monells, Spain; ^3^Department of Animal Nutrition and Animal Health, University of Kassel, Witzenhausen, Germany

**Keywords:** animal health, herbal remedies, efficiency, randomised controlled trials, external validation

## Abstract

This study reflects on the recognised need for more joined-up, high-quality research on phytotherapy that addresses the current societal challenges in finding alternatives to the use of antibiotics. The study applied a multidisciplinary participatory approach in an expert workshop exercise within the FP7 EU IMPRO project. Prior to this study, a literature review was elaborated on research in the field of phytotherapy as applied to farm animals, cooperation between research bodies and initiatives to reduce the use of antibiotics by using phytotherapeutic remedies. The review was delivered to the participants of the workshop so as to receive feedback on it and enrich the discussion. Different expertise, background in research or veterinary practice, and varying positions regarding phytotherapy were the criteria in targeting participants. A structured workshop was subsequently organised, with questions to experts addressing scientific validation of phytotherapy, effective treatment under farm conditions and necessary developments for the future. Challenges identified by the experts were as follows: poor study designs, lack of reproducibility of studies, poor standardisation of products, cost–benefit concerns, lack of veterinarian training and poor data availability. To overcome obstacles, the need for improved study designs for clinical trials was given priority in order to prove the efficacy of remedies and to implement a monitoring system which enables the assessment of the effectiveness of treatments in farm practice. Reflections in this report are intended to be a resource for scientists, policy makers and end users for an effective use of phytotherapy at farm level.

## Introduction

The need to reduce the use of antibiotics in livestock, due to increased concerns over the spread of antibiotic resistance, has raised the interest in phytotherapy (1). However, to the best of authors’ knowledge, no studies of the prescription and use of phytotherapeutic remedies for food-producing animals by veterinarians in Europe are available. A study of the use of phytotherapy reported that 590 plant species, deriving from 102 different plant families, are available to be used for animal treatments in the EU, although only very few are registered for the treatment of livestock (2). Thus, the use of phytotherapeutic products in farm practice is limited. Even in organic agriculture, where phytotherapeutic treatments are promoted by EU Regulation (3), phytotherapeutic remedies are administered to a minor extent in comparison to antibiotics or antiparasitics (4).

Reasons for the low relevance of phytotherapeutic products when treating sick animals are manifold and difficult to grasp. Legal uncertainties are assumed to be one of the most significant obstacles to the use of phytotherapeutic remedies (5). Phytotherapeutic products are not authorised centrally in the EU, but by the national institutions. The number of veterinary herbal medicines approved in the different EU member states, and the extent to which they require veterinary prescription, is not known (6). In addition, the concern and difficulties related to the patenting of herbal medicines have precluded the financial incentives that could be provided to the pharmaceutical industries (7). Nevertheless, it is permitted to sell feed additives containing plant material in different formulations, with claims concerning optimisation of nutrition, support or protection of the physiological conditions, unless they do not contain a claim declaring that they will prevent, treat or cure a disease (8). Due to their easy access, botanical products are primarily sold as feed additives, and they may be used as presumed remedies. These conditions leave farmers and veterinarians to a large extent without scientific and unbiased assessment of their efficacy. Thus, the use of feed additives and non-registered products is often based on the experiences and mental associations of those who administer the products (6).

Previous studies of the efficacy of phytogenic compounds in food-producing animals are highly variable, leading to a general uncertainty regarding the efficacy of phytotherapy (9). So far, *in vitro* and experimental studies have dominated the research work. In general when field studies were carried out under the conditions of commercial farms, the results were not reproduced (5). The use of phytotherapeutic products at farm level might also be related to the uncertainty as to whether professional skills are available to support an effective administration (9).

A large proportion of studies using the same botanical remedies to compare the efficacy of the remedy between the studies were classified as uncertain in a systematic review (6). Clearly, systematic reviews rely strongly on the quality and quantity of primary data (clinical trials), which is low in the field of veterinary herbal medicine. Due to the different methods of preparation and different parts of plants used in herbal extracts, such reviews may not be fully comparable (10) and thus do not provide sufficient scientific guidance.

The first aim of this study was to reflect on challenges and priorities for an effective use of phytotherapy in livestock. For this purpose, a workshop with invited experts from various disciplines was organised. As background to the workshop, a review was conducted, collecting information on current procedures (*lege artis*), performing a systematic literature search of *in vivo* studies on the use of phytotherapy in livestock and distributing a questionnaire in order to assess research collaboration and constraints as viewed by institutions engaged in the field of phytotherapy. The workshop was organised so as to receive feedback on the review as well as to identify important factors influencing the effectiveness and use of phytotherapeutics in research and farm practice in the European context. The second aim of this study is to define a focused future strategic planning on the use and research of phytotherapy in livestock.

## Materials and Methods

### Workshop Design and Selection of Experts

A workshop entitled “Workshop on options and limitations of phytotherapy” was organised with the aim of engaging 12 experts. The selection of candidates as experts from across Europe was based on their scientific and technical expertise and experience. The areas of expertise to be covered were as follows: veterinary medicine and herd health management in practice (two participants), phytotherapy research (2), pharmacology research (1), veterinary epidemiology (2), regulatory agencies (2), evidence-based medicine and animal health and welfare (4). The selection was carried out independently by three researchers involved in the FP7 EU IMPRO project (www.impro-dairy.eu). The evaluation of the expertise of the persons was based on previous publications and experience. When approximately 30 experts were identified, the IMPRO team discussed the composed list of experts. To foster a balance, candidates were classified as neutral, promotors or detractors of phytotherapy prior to the final selection. The list was agreed among the partners of the project. A second round of contacts to candidates and invitation letters were sent out to conform the final number of experts for the workshop. If the response was positive, the following material was provided: an extended agenda with information about the workshop and a consent form. The 12 participants were from different European countries (Great Britain, Germany, Spain, Switzerland, Austria, Sweden, Netherlands and Denmark). Approximately half of the participants were active in the field of phytotherapy, the majority representing researchers but also veterinarians practicing phytotherapy as well as representatives of the industry. All participants were informed that they should keep in mind that they were not representing any organisation. Prior to the workshop, each participant received a copy of the scientific review that forms the Deliverable 9.2 from the FP7 EU IMPRO project (No. 311824), “Report on research projects in the field of phytotherapy, cooperation between research bodies and initiatives to reduce the use of antibiotics by using phytotherapeutic remedies.”

### Procedure

**Figure 1:**
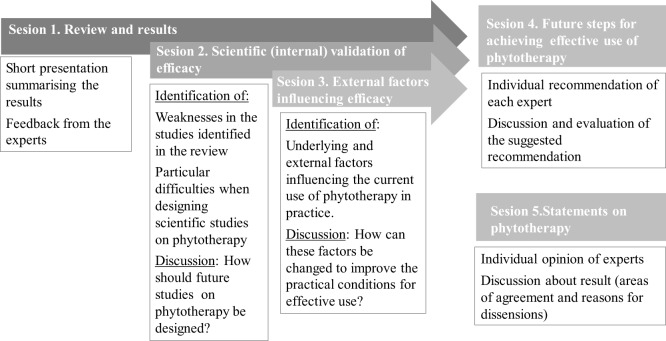
Structure of the workshop and the major topic for each session.

The workshop was arranged in Germany on 12 January 2015. The discussion was guided by a professional coach with moderation and group communication skills Dr. Karina Gregory (Moderatio^®^) also experienced in conflict mediation. In order not to influence the experts, the research group responsible for the review restricted itself to an observing role. The workshop was divided into five sessions (Figure 1). In the first session, an overview of the IMPRO project was presented, followed by a short summary and discussion of the main results and conclusions of the review. In the second session, how studies on phytotherapy should be designed to enable a valid assessment of efficacy was discussed with the participants. This was followed by the third session, in which external factors that influence the effective use of phytotherapy were reflected upon and explored through open discussion on what is necessary to ensure that efficacy is given under varying practical conditions. The fourth session of the workshop was based on the discussions in previous parts to identify future steps for an effective use of phytotherapy. In this session, the experts individually composed suggestions on how the European Commission should proceed on the topic of research and use of phytotherapeutic products in farm animals, especially in order to reduce the use of antibiotics. Finally, an open (non-guided) discussion was scheduled at the end of the workshop in order to capture general feedback.

The moderator introduced the different topics by flipcharts, collected single aspects and opinions of each expert by moderation cards, and supported the dialogue between the experts. The flipcharts were used actively during the workshop and filled in during the discussion by the experts. After the workshop, the charts in combination with the researcher’s notes were used as a protocol. In addition, audio recording was collected as a reference and for clarification during the writing process.

## Results and Discussion

### Research Challenges

Research challenges on what is needed to validate efficacy and what prevents achieving effectiveness highlighted by the experts are described below.

#### Poor Study Design

For a large proportion of the studies discussed at the workshop, the participants supported the statement that the observed effect or lack of an effect within the studies cannot be evaluated appropriately due to poor study design, e.g., poor specification of content or composition of ingredients and dosage of the applied botanical. In both the review and the workshop, it was highlighted that randomised clinical trials are important so as to test the corresponding hypothesis and for establishing the adequate doses. The members of the expert panel agreed that the use of phytotherapy also needs to be evaluated by on commercial farm trials in order to establish their effectiveness in practice.

#### Poor Standardisation and Classification of Products

Poor standardisation of products and limited information regarding the composition of the remedies used cause major problems with regard to reproducibility. Although standardisation is difficult for phytotherapeutic products, as they are complex combinations of several phytochemicals, there are guidelines for presenting content in a standardised way enabling reproducibility. However, the same botanical given to an animal can be categorised as a feed, a feed additive or as a veterinary medicinal product, depending on the purpose for which it is given, and the required documentation between the different categories varies. The necessary requirements of documenting content and standardisation of veterinary medicinal products make it difficult for the manufacturers to protect and patent the product. Consequently, there is a drive for selling promising products as feed additives/single feeds with no indication for treatment or prevention of diseases, but with unspecific health-supporting claims, as the feed sector is less strictly regulated than the medicines sector (6). The participants in the workshop expressed a need for clarification of how some botanical products, such as single feeds and feed additives, should be categorised, and how a standardised presentation of content can be achieved without jeopardising the patentability of the product.

#### Lack of Reproducibility of Studies

Although most studies tend to conclude that phytotherapeutic remedies may have potential, none of the scientific studies have been replicated (5). Even though some investigations under practical conditions in large-scale animal production have shown better responses to treatment than earlier studies under controlled experimental conditions (11, 12), with a higher level of hygiene, the figures are not comparable, since certain conditions (i.e., environmental) on commercial farms were different (13). The workshop participants have indicated that the use of phytotherapy is thus lacking any reproducibility, and the results cannot claim to have a relevant external validity.

#### Cost–Benefit Concerns

Workshop participants considered that it is very expensive to develop new phytotherapeutic remedies, while buying antimicrobials that are already on the market is expected to be less expensive. According to the discussion, this may lead to a more ineffective search for alternatives to the existing antibiotics, as companies do not see it as economically viable to invest in developing new remedies as long as cheaper alternatives are available. There is a directive (14) that established a regulatory framework for traditional herbal medicinal products to meet specific and appropriate standards of efficacy, safety and quality, and this framework is quite costly for manufacturers. Consequently, the approvals of medicinal phytotherapeutic products are associated with high costs, but combined with small profits from sale, discourage companies from developing these types of products.

#### Lack of (Veterinary) Training

There was disagreement among the participants with respect to what extent farmers and lay persons are able to diagnose and follow-up treatments. However, all participants saw that clinical veterinarians play an important role in supervising, advising and educating the farmer, as well as taking the responsibility for the success of treatment and the fact that the issues of animal welfare and food safety are appropriately taken into account. However, how to deal with this issue is interpreted somewhat differently in different countries. For example, in Sweden, it is not generally considered *lege artis* for veterinarians to prescribe phytotherapeutic remedies that are not approved veterinary medical products, while veterinarians in Germany more frequently use these types of products (6).

Due to the possible risks for animal health and welfare, only individuals with appropriate education and training should make use of phytotherapy. The possibilities for education in the subject of phytotherapy vary considerably between countries (15). In a study of Fernández González et al. (9), veterinarians or feed advisors from France, Germany, Netherlands and Spain using phytotherapy were interviewed. Even those who had used phytotherapeutic products for longer time periods had little or no formal education on this matter. Currently studies of the prescription of phytotherapeutic remedies for food-producing animals by veterinarians in Europe are not available. Nor is there a generally approved specialisation for veterinary phytotherapy/herbal medicine in the European Union. Currently, the study of botanicals mainly has a place in the subject of toxicology within the veterinary curriculum (6). Nowadays, little information regarding the administration to animals is available, and how to establish effective dosage is largely missing from scientific literature and textbooks (2). The experts agreed that the possibility for interested farmers and veterinarians to gain more knowledge is limited and varies between countries in the European Union. Tradition, lack of knowledge and education in many parts of Europe is limiting the use of these treatment methods (16).

#### Poor Data Availability

All participants in the workshop noticed that monitoring and control of effectiveness of all kinds of treatments in veterinary practice in Europe is insufficient. Under these circumstances, it is very difficult to prove effectiveness of phytotherapy, as well as of conventional treatments, in practice (6).

### Further Steps for Improving Research and Use of Phytotherapy

**Figure 2:**
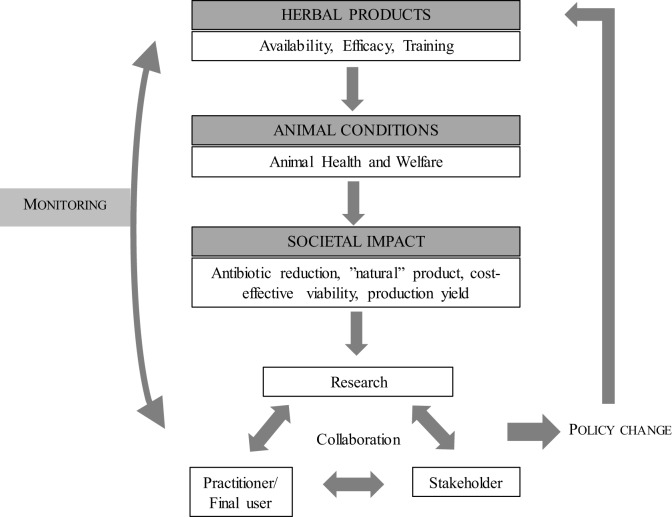
Disciplines and impact flows related with the main challenges in phytotherapy.

The participants in the workshop provided recommendations on concrete next steps and operational guidance belonging to different discipline areas and impacting on different actors (Figure 2).

#### Improving Quality of the Study Design

According to the participants, the use of randomised clinical control trial study design is also applicable to phytotherapeutic products, although they may require some adaptations based on the particular substance, use and expected efficacy. The experts discussed the importance of choosing products to treat diseases and calculating doses based on knowledge of practitioners, and other experience bases when designing trials. They concluded that relying on experience might also give an indication of which effect of the botanical could be expected and support this so as to adapt the study design. Applying analytical methods identified by the experts, such as metabolomics, could help to better identify the ingredients, and ensure standardisation and safety of the remedies. Recent development of high-throughput and “omics” technologies might accelerate studies of the mechanisms, underlying phytogenic compounds’ functions and, therefore, guide the effective use of the compounds (5).

#### Development of Specific Guidelines

In order to assess effectiveness in practice, the participants discussed the need for field trials in which farm-specific conditions are taken into account. Different farms have different conditions that add to the complexity when designing studies to measure the effectiveness of remedies. It would, therefore, be helpful if specific guidelines and templates for designing controlled experiments studies on phytotherapeutic remedies were developed with established recommendations for dosage and effective use for the target species. Guidelines have to be formulated on the standardisation of contents and on safety analysis for future studies to ensure harmonisation.

#### More Education and Training for the Next Generation of Veterinarians

Reintroducing phytotherapy into the curriculum of European veterinary education was discussed as an option although associated with concerns like adaptation to international accreditation schemes. As phytotherapeutic products are currently applied at different levels (as feed material/additive by farmers or nutritionists; as a remedy or metaphylaxis/prevention–prescription by the veterinarian), each application requires adequate knowledge and, if products are used, there should be high-quality courses available for interested farmers and veterinarians. Experiences and knowledge might be gained in the future from the currently existing specialisation for veterinarians in Switzerland and Austria in veterinary phytotherapy.

#### Adapting the Legislation to Better Describing Phytotherapeutic Remedies As a Class of Products and Harmonise with Human Regulations

An adaptation of the legislation would potentially make it possible to register traditional veterinary herbal medicinal products through a simplified registration procedure or support the registration as health-supporting feed supplements (zootechnical feed additives). Some experts suggested adapting the requirements for safety assessments to the specific circumstances surrounding botanicals that have been used for a long time and that are included in European monographs for human use. One suggestion in order to simplify the use was to follow Switzerland and exclude phytotherapy from the cascade principle.^1^ At the EU level, a framework should be developed for the regulation of substances that reduce the need for or use of antimicrobials to solve the conflict arising by the definitions of either a veterinary medicinal product or a feed additive, considering the possible use of specific claims (17).

#### Establishing a European Monitoring System for Production Diseases and Treatments

This was a main recommendation all experts agreed upon. There is a need to establish a monitoring system for the efficacy of both antibiotics and herbal medicines by monitoring the prevalence of production diseases, the frequency of treatments and also of pathological findings at abattoirs across Europe. This would provide essential possibilities for evaluating therapeutic success and the consecutive control of effects. A monitoring system would also make it possible to follow the impacts of health improving measures (like herbal treatments, but also other measures like increased biosecurity) on specific farms over time, taking into account different farm characteristics, management routines, feeding regimes, etc. Given the ongoing obstacles to the availability of data, diagnostic laboratories appear to provide the most readily available data sources for syndromic surveillance in relation to production diseases (18).

#### Making the Development of New Products and Production of Phytotherapeutic Remedies Attractive in Terms of Cost-Effectiveness for Manufacturers

Incentives for companies to perform clinical trials with herbal medicines should be established through a period of data protection or protection of composition of treatments. The experts suggested a period of 5 years. It is important to establish research conditions for non-patentable common goods (e.g., pure drugs and easily available herbs), based on identified and standardised ingredients.

For the development of new products, research supported by external funding has to address standards of quality, safety and efficacy in parallel with the development of specific guidelines and protocols that help to produce appropriate study designs and product standardisation. Another request is to provide a post-authorisation monitoring system for herbal products so as to be able to assess to what extent they are reliable as an alternative to antibiotics.

More financial support is needed, apart from that by pharmaceutical companies, in the view of experts. Similar results were obtained in the questionnaire survey of the review, in which insufficient support by funding bodies was identified as a main constraint in research work (6). The workshop participants recommended focusing such funding on the proof of efficacy with respect to specific indications where antibiotics have been widely used, such as in the case of prophylactic use for the most prevalent disease problems in pig and poultry.

#### Strengthen Collaboration in Research

The participants in the workshop also recommended the support of research comprising internal (study design) and external (efficacy at farm) validation. Stakeholders, e.g., farmers and their veterinarians, should be able to identify and ask important (research) questions, the researcher would acquire the required knowledge using trials with an appropriate study design, and would evaluate scientific results, while the stakeholder, e.g., practitioner, would apply and assess the results on the farms by monitoring and reporting them back. This would correspond with the five steps according to the cycle of evidence-based veterinary medicine: Ask, Acquire, Appraise, Apply, and Assess (19).

The major outputs of the workshop were in line with those described in a recent event at the EMA and EFSA Joint Scientific Opinion on measures to reduce the need to use antimicrobial agents in animal husbandry in the European Union, and the resulting impacts on food safety (RONAFA) (17). In this report, developing new effective antimicrobials or alternatives for treatment, promoting research and innovation as well as improving communication, education and training were identified as key areas to be addressed.

## Conclusion

The major obstacles to overcoming an inappropriate use of the phytotherapy are as follows: (1) the conflict between missing results concerning efficacy and the interests of the farmers to use feed additives without prescriptions by the veterinarian; (2) the request for better education of veterinarians when, on the other hand, the prerequisites of evidence-based efficacy are not available; and (3) the conflict between commercial interests and the tasks of the authorities to ensure animal welfare and food safety. Only coordinated and sustained efforts (including long-term funded research) by all players in the livestock sector as well as developers of regulatory framework will allow possibilities to release the potential therapeutic ability of phytotherapy.

## Author Contributions

IB-P wrote the manuscript and CG have made substantial contribution to conception and acquisition of data. L-MT provided valuable expertise in the writing process. AS and UE assisted in the conceptualisation of the manuscript and the review. IB-P checked the references and acted as corresponding author. All the authors have read and approved the manuscript.

## Conflict of Interest Statement

The authors declare that the research was conducted in the absence of any commercial or financial relationships that could be construed as a potential conflict of interest.

## Footnotes

^1^Cascade principle: Directive 2004/28/EC clearly states that veterinarians should primarily use a product approved for the particular condition and the particular species. If botanicals are not approved, this means that veterinarians are not allowed to prescribe them for treatment and prophylaxis of diseases.

## References

[1] Parreira P Brás T Ramos PA Duarte MF Bioactives against Superbugs: using phytotherapy to counteract the drug resistance burden in the 21st century. In: éndez-Vilas AM, editor. The Battle Against Microbial Pathogens: Basic Science, Technological Advances and Educational Programs. Badajoz, Spain: Formatex Research Center (2015). p. 109–16.

[2] Mayer M Vogl CR Amorena M Hamburger M Walkenhorst M. Treatment of organic livestock with medicinal plants: a systematic review of European ethnoveterinary research. Forsch Komplementmed (2014) 21(6):375–86. 10.1159/000370216 25592949

[3] European Commission. Commission regulation (EC) No 889/2008 of 5 September 2008 laying down detailed rules for the implementation of Council Regulation (EC) No 834/2007 on organic production and labelling of organic products with regard to organic production, labelling and control. Off J Eur Union (2008) 250:1–115.

[4] Rymer C Vaarst M Padel S Future perspectives for animal health on organic farms: main findings, conclusions and recommendations from SAFO Network. Proceedings of the 5th SAFO Workshop Odense (2006). 145 p.

[5] Yang C Chowdhury MK Huo Y Gong J. Phytogenic compounds as alternatives to in-feed antibiotics: potentials and challenges in application. Pathogens (2015) 4:137–56. 10.3390/pathogens4010137 25806623 PMC4384076

[6] Tamminen L-M Blanco Penedo I Fernandez Gonzalez C Sundrum A Report on Research Projects in the Field of Phytotherapy Cooperation between Research Bodies and Initiatives to Reduce the Use of Antibiotics by Using Phytotherapeutic Remedies (2016). IMPRO Project Number: 311824.

[7] Calixto JB. Efficacy, safety, quality control, marketing and regulatory guidelines for herbal medicines (phytotherapeutic agents). Braz J Med Biol Res (2000) 33:179–89. 10.1590/S0100-879X2000000200004 10657057

[8] European Commission. Regulation (EC) No 767/2009 of the European parliamentand of the council of 13 July 2009 on the placing on the market and use of feed, amending European Parliament and Council Regulation (EC) No 1831/2003 and repealing Council Directive 79/373/EEC, Commission Directive 80/511/EEC, Council Directives 82/471/EEC, 83/228/EEC, 93/74/EEC, 93/113/EC and 96/25/EC and Commission Decision 2004/217/EC. Off J Eur Union (2009) 229:1–28.

[9] Fernández González C Blanco-Penedo I Velarde A Report on the Preconditions for an Effective Use of Phytotherapy in Pig and Poultry Production (2016). IMPRO Project Number: 311824.

[10] Izzo AA Hoon-Kim S Radhakrishnan R Williamson EM. A critical approach to evaluating clinical efficacy, adverse events and drug interactions of herbal remedies. Phytother Res (2016) 30:691–700. 10.1002/ptr.5591 26887532

[11] Castillo M Martin-Orue SM Roca M Manzanilla EG Badiola I Perez JF The response of gastrointestinal microbiota to avilamycin, butyrate, and plant extracts in early-weaned pigs. J Anim Sci (2006) 84(10):2725–34. 10.2527/jas.2004-556 16971574

[12] Kirchgessner M Windisch W Roth FX Effect of avilamycin and tylosin on the metabolizable energy in growing and finishing pigs. Arch Anim Nutr (1995) 48:63 10.1080/17450399509381828 8526732

[13] Franz C Baser KHC Windisch W Essential oils and aromatic plants in animal feeding – a European perspective. A review. Flavour Frag J (2010) 25(5):327–40. 10.1002/ffj.1967

[14] European Commission. Directive 2004/24/EC of the European parliamentand of the council of 31 March 2004 amending, as regards traditional herbal medicinal products, directive 2001/83/EC on the Community code relating to medicinal products for human use. Off J Eur Union (2004) 136:85–90.

[15] Vaarst M Martini A Bennesgard TW Hecktoen L Approaches to the treatment of diseased animals. In: Vaarst M Roderick S Lund V Lockeretz W, editors. Animal Health and Welfare in Organic Agriculture. Oxford, UK: CABI Publishing (2004). p. 279–307.

[16] Martini A Polidori R Lorenzini G Lotti C Whittaker A Efficiency and costs of homeopathy and phytotherapy in an organic dairy farm. New Medit (2012) 11(4):42–5.

[17] EMA (European Medicines Agency) and EFSA (European Food Safety Authority). EMA and EFSA Joint Scientific Opinion on measures to reduce the need to use antimicrobial agents in animal husbandry in the European Union, and the resulting impacts on food safety (RONAFA). EFSA J (2017) 15(1):4666 10.2903/j.efsa.2017.4666 PMC7010070 32625259

[18] Dórea FC Sánchez J Crawford WR Review, veterinary syndromic surveillance: current initiatives and potential for development. Prev Vet Med (2011) 101(1–2):1–17. 10.1016/j.prevetmed.2011.05.004 21640415

[19] Dean R Evidence-based veterinary medicine: Experiences, progress and new horizons. In: Knowledge RCVS, editor. EBVM – First International Evidence-Based Veterinary Medicine Network Conference Windsor, UK: EBVM Network (2014). p. 64–5.

